# Assessment of the direct medical costs of diabetes mellitus and its complications in the United Arab Emirates

**DOI:** 10.1186/1471-2458-10-679

**Published:** 2010-11-08

**Authors:** Fatma Al-Maskari, Mohammed El-Sadig, Nicholas Nagelkerke

**Affiliations:** 1 Department of Community Medicine, Faculty of Medicine & Health Sciences, United Arab Emirates University, Al-Ain, P.O. Box: 17666, United Arab Emirates

## Abstract

**Background:**

Diabetes mellitus (DM) is a major health problem in the United Arab Emirates (UAE) and is well recognized as a major and increasing burden to the country's resources due to its severe, long term debilitating effects on individuals, families and the society at large. The aim of the study was to estimate the direct annual treatment costs of DM and its related complications among patients in Al-Ain city, UAE.

**Methods:**

A sample of 150 DM patients were enrolled during 2004-2005, and their medical costs over the ensuing 12 months was measured, quantified, analyzed and extrapolated to the population in Al-Ain and UAE, using conventional and inference statistics. The costs were converted from UAE Dirhams to US Dollar, using the official conversion rate of US$ (1 USD = 3.68 AED).

**Results:**

The total annual direct treatment costs of DM among patients without complications in Al Ain-UAE, was US $1,605 (SD = 1,206) which is 3.2 times higher than the per capita expenditure for health care in the UAE (US$ 497) during 2004 (WHO, 2004). However, this cost increased 2.2 times with the presence of DM related complications for patients with microvascular complications, by 6.4 times for patients with macrovascular complications and 9.4 times for patients with both micro and macrovascular complications. Likewise, the annual direct hospitalization costs of DM patients increased by 3.7 times for patients with microvascular complications, by 6.6 times for patients with macrovascular complications and by 5 times for patients with both micro and macrovascualr complications. Overall, costs increased with age, diabetes duration and were higher for patients treated with insulin compared to those treated with oral hypoglycemic agents or with diet control only.

**Conclusions:**

DM direct treatment costs increased with the presence and progression of chronic DM related complications. Hospitalisation costs constituted a large proportion and were increasingly higher with the presence and progression of DM related complications. To reduce the impact on healthcare resources, efforts should be made to prevent progression to DM complications, by implementing guidelines for diabetes care, screening for complications and better management.

## Background

Diabetes Mellitus (DM) is a major cause of morbidity, disability and mortality worldwide [1]. In addition, the economic burden on patients and society in the form of direct and indirect costs is enormous [1]. The IDF projections show that the health care expenditures on diabetes will account for 11.6% of the total healthcare expenditure in the world in 2010 [1]. A recent study in the U.S. estimated that annual costs in 2007 exceeded US $174 billion [2]. A similar study in Italy showed that the direct costs of healthcare in 2006 were €2,589 per DM patient, compared to €1,682 for matched people without diabetes [3]. In Europe, the CODE-2 study showed that more than 10 million people with type 2 diabetes across eight European countries cost over EUR 29 billion in 1999 M [4].

The prevalence of diabetes in the United Arab Emirates (UAE) is reportedly among the highest in the world [1]. A population survey in Al Ain, UAE, in 2000 estimated the prevalence of DM at 25% among nationals (UAE citizens) and 19% among non-nationals (other GCC countries and expatriates) [5]. Another recent study in the UAE showed that 19% of diabetic patients suffered from DM retinopathy, 35% from DM neuropathy, 12% from peripheral vascular disease, 14% from coronary artery disease, 4% from cerebro-vascular disease, 35% from hypertension and 31% from dyslipidaemia [6,7]. The Ministry of Health in the UAE also reported that DM constitutes the 6th leading cause of death in the population [8].

Nevertheless, with only three studies from countries in the region (Iran, Tunisia and Egypt) there is a dearth of information on the economic impact of DM in the Middle East [9-12], especially the Arabian Peninsula, where no specific studies were carried out. WHO estimates for the per capita expenditure on health care in the region amounted to US$ 497 for UAE during 2004 [13]. To fill this gap we attempted to estimate the economic impact of DM in Al-Ain district, UAE. Al Ain district is located in the interior of Abu Dhabi Emirate and is the second largest city after Abu-Dhabi with population of (approximately 500,000). This study aims to estimate the overall patient treatment costs of DM in this part of the UAE; including the direct management costs of the disease and its related chronic macro and micro-vascular complications.

The earliest studies that attempted to measure the economic impact of DM used the Cost of Illness (COI) approach. This approach, originated by Mushkin [14], Weisbrod [15], Rice [16], and others in the early 1960s, estimates the direct economic burden of diseases, with the single objective of priority setting in health care planning. However, subsequent DM cost studies were more complex [17] and considered costs due to co-morbidity making use of either existing data, e.g. routine data collected by the health care system and cost projections from previous studies [17], or collected new data, e.g. using surveys of the DM population [18]. Most recent DM cost studies are based on either national diabetic population surveys or on routinely collected data that only attribute medical management costs to DM when the disease is listed as the (primary) diagnosis for a health care visit, disability or cause of death [18]. Thus, many of those studies tend to underestimate DM costs as they ignore costs caused by other illnesses in which DM is a contributory factor. The ideal approach would be to assess all costs that would have been averted ("avertable" costs) in the counterfactual situation in which DM would have been eliminated [19]. However, this is methodologically complex, as such avertable costs are not directly observable, and empirical studies face major challenges such as confounding [15]. While advanced statistical methods are often used to overcome such problems it is not clear to what extent findings are sensitive to the assumptions that enter into such calculations [16].

## Methods

The view point of this study is that of the health care system, i.e. it attempts to assess the direct treatment costs imposed by DM and its related complications on the health care sector of the country. Indirect costs such as productivity losses resulting from lost work days, which may be even larger than the direct medical costs, were not considered. To estimate these costs per individual patient (unit), we explored the treatment profiles of a (cross-sectional) sample of DM patients over a one year period. Clinical information about type of diabetes, diabetes related complications and co-morbidity, severity and treatment profile of patients were based on diagnosis and judgements by treating doctors.

### 1. Setting

The study was carried out at the two major hospitals, Tawam and Al-Ain, serving 75% of patients in the Eastern District of Abu Dhabi emirate (Al-Ain region). The health care system in the region at the time was organised along the lines of the conventional health care system, i.e. primary health care, through which 18 clinics provide basic health care to DM patients, and secondary and tertiary care to patients through the two referral hospitals in the region: Tawam and Al-Ain. Provision of health care at the time was administered through a health insurance scheme for both nationals and non nationals. Patients of all nationalities used to have equal access to health care services with exception of non health card holders. Patients with chronic complications such as DM had equal right to access treatment at both hospitals. At present, health care is free for local citizens and effective from 2006, all non local residents of Abu Dhabi are covered by a new obligatory comprehensive health insurance scheme.

### 2. Study design and selection of participants

The study was part of a general cross-sectional survey of DM patients carried out earlier to assess and establish the prevalence of DM complications among diabetic patients in Al-Ain District, UAE [6,7]. The sampling frame of the study included all UAE and non-UAE diabetic patients of all ages and both genders, attending the outpatient clinics of the two main referral hospitals (Al-Ain and Tawam hospitals) for DM care. In the absence of diabetes registries, patients were randomly selected from the lists of clinic appointments. The study period (for cost calculations) was the year preceding this index visits. Sample sizes were based on the requirement that prevalence estimates should have an (absolute) standard error of no more than 0.02 (2%). Accordingly, a sample size of 625 was calculated and approached, out of whom 513 (82%) agreed to enrol, of whom 150 (29.3%) patients were enrolled from the two diabetics clinics of the two hospitals. The study was approved by the Ethics Committee of the Faculty of Medicine and Health Sciences of the UAE University.

### 3. Data collection and definitions

Although a vast majority of DM patients receive regular treatment at primary health care clinics in Al-Ain district, the current system of primary medical care in Al-Ain is not based on continuity of care and accountability but rather on rapid access without appointment to any physician available. Chronic disease clinics that have been recently implemented in three primary centres may have an impact on quality of care but due to logistical reasons (data completeness and accessibility), only patients attending outpatient clinics at Al- Ain and Tawam hospitals were included in the study.

Consenting hospital patients were asked to complete an interviewer administered questionnaire on frequency of initial and follow up visits, hospital admissions and all medical support causally related to DM management during the past 12 months to validate hospital and clinic data. Clinical data for patients were retrieved from their medical records at the two hospitals.

It was not always possible to distinguish clearly between types 1 and 2 diabetes from the medical records alone and for that we opted to use the terms "insulin treated diabetes", and "non-insulin treated diabetes" to classify DM patients. The following sequelae of chronic diabetic cardiovascular complications were considered for analysis: (1) Macro-vascular complications: acute myocardial infarction, angina pectoris, stroke, and non-traumatic amputation and foot ulcers; (2) Micro-vascular complications: various levels of nephropathy (i.e. microalbuminuria, clinical proteinuria and chronic renal failure), retinopathy (i.e. background and proliferative retinopathy, macular edema, cataract and blindness) and peripheral neuropathy. The diagnosis of various chronic complications of DM was based on medical records.

To assign costs to the care received by patients, the study used the official list of charges/rates for patients not covered by health insurance at the time (the official rates for non health card holders). Those charges/prices were allegedly based on approximate real costs; i.e. the rates which, if charged to all individual patients, would have covered the actual costs incurred to provide these services without significant profits and/or subsidies. The average unit costs per patient were estimated by using expert opinion (consultants and doctors from participating hospitals) to distinguish and verify from the files of individuals patients, health care consumption by individual patients (including accommodation, pharmaceuticals, laboratory, nursing costs etc), likely received for DM related conditions and that for other- unrelated-reasons. Costs for DM related conditions were then assigned by calculating the charges that non-card holders would have paid for such health care services. These unit costs were then extrapolated to all patients whether he/she had actually paid for them or not (UAE nationals are automatically insured against all medical costs). To comprehend the combined cost impact of DM complication on health care resources, the study calculated and compared the treatment costs of DM patients without complications to those of patients with micorvascualr complications, those with macrovasualr complications and those with both, macro and microvascualr complications.

### 4. Statistical Analysis

Data entry and analysis was performed using the statistical software package (SPSS version 13). Cost estimates are presented as point estimates with associated standard deviations. The study used conventional methods of statistical inference and hypothesis testing. The Chi-square test and the t-test were used, where appropriate, to test for significance (*p *< 0.05 is considered statistically significant). Linear regression, with logarithmically transformed costs as the dependent variable, was used to estimate the simultaneous effect of various patient characteristics on costs. A logarithmical transformation was used, as patients characteristics were believed to act multiplicatively on costs rather than additively. As random selection of patients attending the clinic may lead to size-biased sampling (those attending the clinic more frequently are oversampled), the number of visits of a patient to the clinic was treated as a "design" variable and either used (inversely) as a weighting factor, or included as a covariate in our regression/covariance analyses.

## Results

### 1. Demographic characteristics

A total of 150 DM patients were enrolled; 63% from Tawam hospital and 37% from Al-Ain hospital. Of the total sample 67% were males, 48% were UAE nationals, 7% were citizens from other Gulf (GCC) countries, 30% were resident Arabs from other countries and 15% were expatriate Asians living and working in the UAE. Thirty three percent were above sixty years. The mean age was 55.6 years (SD: 13.3: range 14-85) and 46% were illiterate (Table 1).

**Table 1:** Average Annual Treatment Costs in US $ by Patient Demographic Characteristics and Health Care Centre in the UAE, during 2004 (N = 150)

Patient Socio Demographic Characteristics		N	%(95% C.I)	Mean $ Annual Cost	Std Deviation
**Participant Health Care Centre**	Tawam hospital	95	63.3 (55.6-71.0)	5,262	5,980
	Al-Ain Hospital	55	36.7 (29.0-44.4)	5,096	5,779
					
**Sex**	Male	101	33% (25.5-40.5)	5,470	6,028
	Female	48	67% (59.5-74.5)	4,646	5,609
					
**Education level**	Illiterate	55	46% (38.0-54.0)	5,602	5,747
	Primary school	48	27% (20.2-34.4)	4,473	6,388
	Secondary school	20	19% (9.0-20.4)	4,583	4,880
	Completed University	26	6% (6.8-17.2)	6,078	6,567
					
**Nationality**	UAE	50	48% (40.4-56.0)	5,645	5,966
	GCC	4	7% (2.9-11.1)	7,025	5,532
	Other Arabs	59	30% (22.7-37.3)	4,417	6,016
	Asians	35	15% (9.3-20.7)	4,304	5,405
					
**Occupation**	Housewife or Retired	70	57.0% (49.1-64.9)	5,493	5,876
	Employee	42	22.8% (16.1-29.6)	5,650	6,759
	Labourer	33	16.8% (10.8-22.8)	4,334	5,260
	Student	3	3.4% (0.5-6.3)	2,350	976
					
**Age Group**	<20 yr	1	0.4%	2,610	1,492
	20 - 39 yr	10	7% (3.7-12.3)	1,328	1,331
	40 - 60 yr	99	59% (51.1-66.9)	4,759	5,939
	>=61 yr	38	33% (25.5-40.5)	6,175	6,647

### 2. Clinical History of the Sample Population

Fifty-seven percent of the sample populations were 'non insulin treated' DM patients. Forty- percent had the disease for more than 11 years and 43% were 'insulin treated' DM patients (Table 2).

**Table 2:** Average Annual DM Treatment Costs in US $ by Patient Clinical Characteristics and Health Care Provider in the UAE during 2004 (N = 150)

Patient Clinical Characteristics		N	% (95% C.I)	Mean $ Annual Cost	Std Deviation
**Type of DM**	insulin treated	55	43% (35.1-50.9)	6,778	6,651
	non insulin treated	94	57% (49.1-64.9)	3,995	4,941
					
**Mode of Diagnosis**	Incidental	36	13% (7.6-18.4)	8,055	7,276
	Screening	26	23% (16.3-29.7)	3,538	3,811
	Symptomatic	88	63% (55.3-70.7)	5,213	6,021
					
**Family History of DM**	Present	97	65.1% (57.4-72.8)	5,144	6,015
	Absent	52	34.8% (27.2-42.4)	4,319	5,978
					
**Current health care provider**	Primary Health Clinic	43	28.6% (21.3-35.9)	4,077	5,007
	Hospital Diabetes specialist	70	46.6% (38.6-54.6)	6,117	6,315
	Hospital ER	1	0.6% (-0.6-1.8)	16,923	0
	Private clinic	34	22.6% (15.9-29.3)	3,576	5,382
	Other health care provider	2	1(-0.5-2.5)	7,139	6,059
					
**Duration of DM**	<=5 yrs	40	26% (19.2-33.2)	3,004	3,946
	6 - 10 yrs	40	26% (19.2-33.2)	4,598	5,231
	11 - 20 yrs	60	40% (31.8-47.4)	6,191	6,605
	>=21 yrs	9	8% (3.7-12.5)	9,783	6,573

### 3. Patients' Treatment and Care Profile for DM and its related complications

Patients' attendance to the two clinics during the study period varied largely: 26% of patients visited the clinic 7-8 times, 37% paid 3-6 visits, 17% paid 1-2 visits and only 8% did not visit the clinics at all during the previous year. Fifty-one percent of the population had a test for HbA_1C _in the previous year, 91% took a test for fasting blood sugar, 69% for fasting lipid and 69% for renal function, 63% had urinalysis, 38% were tested for urine microscopy, 49% for microalbuminuria and 29% had a 24-hour collection for proteins and creatinine clearance during the past year (Table 3).

**Table 3:** Treatment Profile of DM Patients attending PHC and Hospital Clinics in the UAE during 2004 (N = 150)

Variable	N	Percent
**Annual Patients' Visits to PHC or DM Clinics**		
1-2 visits	25	17%
3-6 visits	55	37%
7 - 11 visits	39	26%
12 or more visits	18	12%

**Laboratory tests**		
HbA_1c_	72	50%
Fasting blood sugar	136	91%
Fasting lipid profile	103	69%
Renal function test	104	69%
Urine analysis	55	37%
Urine microscopy	94	63%
Microalbuminuria	73	49%
24 Hour collection for proteins and creatinine clearance	43	29%
	
**Hospitalization due to DM complications in the past year**		
Never	113	76.6%
1 time	22	14.6%
2-3 times	11	7.3%
4-5 times	4	2.6%

**ER visits due to DM in the past year**		
Never	81	54%
1 time	29	19.3%
2-5 times	30	20%
More than 5 times	8	5.4%
Don't know	2	1.3%

The analysis of the clinical treatment profile of DM patients during the study period showed that 54% percent had been admitted to hospital at least once for DM related complications, 27% were admitted twice, 9% were admitted three times or more and only 10% had not required any hospital admission. During the study period, 19% of the sample patients were admitted at least once to the emergency room (ER) due to DM complication, while 20% were taken to the ER for about 2-5 times (Table 3).

Concomitant risk factors, among patients with DM related complications, were very common: 53% had high cholesterol levels, out of which 44% were on lipid lowering agents. Fifty-eight percent (95% CI: 50.1-65.9%) had hypertension, out of whom 54% were on antihypertensive treatment. Eighty-one percent (95% CI: 75.1-87.5%) had symptomatic peripheral neuropathy, 35% had a history of foot or leg ulcers, and 11% had a history of amputation due to diabetes.

The analysis of the clinical history of the sample population showed that 69% underwent an ECG examination during the study year. Sixty percent reported a history of heart disease, 28% reported a history of heart attack and 15% had a history of heart operation(s). Twenty percent had a history of stroke of whom, 90% received treatment in the hospital ward and only 9% had physiotherapy. Thirty-three percent of stroke patients had spent at least one week in the hospital following stroke attack and 66% spent more than a week (Table 4). Fifty-eight percent (95% CI: 50.2-66%) had diabetic retinopathy; out of whom 42% were tested for visual acuity, while 46% had eye pressure measurement and 40% had retinal imaging during the preceding year. Of the total sample 17% had a history of laser treatment for retinopathy and 21% had (ever) undergone cataract extraction. Thirty-four percent had diabetic nephropathy, out of which 14% experienced chronic renal failure. Of those who had renal failure, 71% were on haemodialysis and 29% had undergone kidney transplantation (Table 4).

**Table 4:** Cardiovascular Risk and Treatment Profile of DM Patients in the UAE during 2004 (N = 150)

Variable	Percent
**Dyslipidemia**	
Presence of dyslipidemia (High Cholesterol)	53.3%
Use of lipid lowering agents	44%
	
**Hypertension**	
Use of antihypertensive agents	54%
	
**Foot Complications**	
Prevalence of symptomatic neuropathy	81.3%
Prevalence of leg or foot ulcer	35.3%
Amputation	11.3%
	
**Coronary artery disease**	
ECG examination	69%
Heart Disease	60%
Heart Attack	28%
Heart Operation	15%
	
**Cerebro-vascular disease**	
Prevalence of Stroke	8%
Observation and treatment at hospital ward	90%
Treatment with physiotherapy	9.1%
	
**Duration of treatment for stroke at hospital ward**	
1 week or less	27%
2 weeks or more	73%
	
**Diabetic Retinopathy**	
Prevalence of Retinopathy	58%
Patients undergone visual acuity test	42%
Patients had eye pressure measurement	46%
Patients undergone retinal imaging	40%
Patients undergone laser treatment	16.7%
Patients undergone operation for cataract extraction	20.7%
	
**Diabetic Nephropathy**	
Prevalence of diabetic nephropathy	34%
Prevalence of chronic renal failure	13.6%
Patients with chronic renal failure who had undergone hemodialysis	71%
Patients with chronic renal failure who had undergone kidney transplantation	29%
	
**Self reported general assessment of Health Status**	
poor health	61%
good health	39%

The assessment of the overall health status showed that 61% reported to have suffered poor health during the past month (before the survey) while 39% reported to have good health status (Table 4).

### 4. Direct Treatment Costs of Diabetes Mellitus

Table (1) shows the unit management costs of diabetes by various patient characteristics; male treatment costs were higher, compared to females (mean $5,470 vs. $4,646) and treatment costs were also higher among patients from neighbouring Gulf Council countries (GCC) (Table 1). The annual mean treatment cost of a GCC patient amounted to $7,025 compared to $5,645 per UAE patient or $4,304 per Asian patient. DM treatment costs among patients also increased with increasing age (Table 1). Young patients, aged 20-39 years, had the lowest annual mean treatment cost viz. $2,610 compared to $6,175 for those aged 61 years and above (Table 1). Likewise, DM management costs increased with duration of disease. Those who had the disease for less than 5 years had a mean unit treatment cost of $3,004, compared to $9,783 for those who had DM for more than 20 years (Table 2).

Management costs of DM also varied with the mode of diagnosis. Patients diagnosed incidentally had the highest annual mean cost amounting to$8,055 compared to only $3,585 for those diagnosed by screening, perhaps due to the medical conditions that prompted the incidental diagnosis (Table 2).

**Figure 1:**
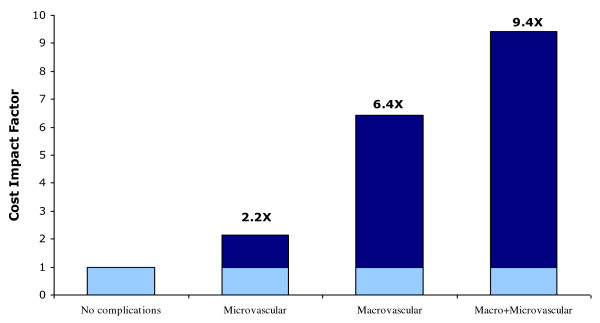
**Effect of DM Complications on the Average Cost per Patient in the UAE during 2004 (n = 150)**.

**Figure 2:**
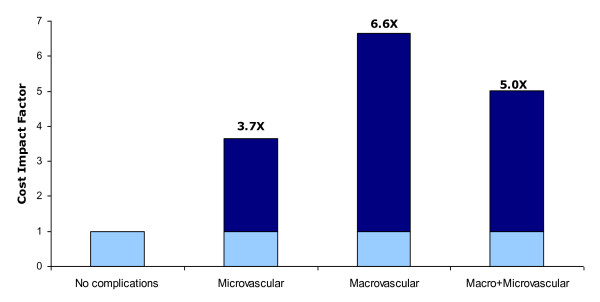
**Effect of DM Complications on Hospitalization Cost per Patient in the UAE during 2004 (n = 150)**.

The annual mean treatments cost for patients on diet control and without complications was US$ 1,605 (SD = ±$1,206) (Table 5). However, the presence of DM complications substantially increased the average patient treatment costs (Table 5). For example, the annual mean treatment cost of patients on insulin is US$ 7,086 (SD ± $ 6425) and for those with hypertension the annual mean cost was 6,210 (SD ± $6,132) (Table 5). Annual treatment costs of patients with microvascular complications were 2.2 times higher than the costs of those without complications (Table 6 & Figure 1). DM treatment costs of patients with macrovascular complications were 6.4 times higher than those without complications and the costs of those with both micro and macrovascular complications were 9.4 times higher. Hospital costs constituted a large proportion of overall treatment costs (Table 6 & Figure 2).

**Table 5:** Average Annual Treatment Costs in US $ by DM Patient Complications in the UAE during 2004 (Weighted, N = 150)

Patient Characteristics		N	%	Average (Mean) $ Cost	Std. Deviation
No DM related Complications	Yes	47	31%	1,605	1,206
					
On insulin	Yes	59	39.5%	7,086	6,739
	No	90	60.5%	3,760	4,696
					
On oral hypoglycemic agents	Yes	98	65.7%	4,262	5,043
	No	51	34.2%	7,079	6,975
					
DM Eye related complications	Yes	76	51%	6,445	6,590
	No	57	38.2%	3,298	4,057
	Don't know	14	9.4%	5,957	6,601
					
Heart attack	Yes	46	30.8%	5,557	8,230
	No	103	69.2%	3,258	2,955
					
High cholesterol	Yes	77	51.6%	6,532	6,786
	No	60	40.2%	3,680	4,260
	Don't know	11	7.4%	3,681	4,105
					
Hypertension	Yes	83	55.7%	6,210	6,432
	No	66	44.3%	3,807	4,748
					
Stroke	Yes	7	15.2%	7,261	5,073
	No	39	84.8%	6,454	7,368
					
Foot or leg ulcer or sore not healed for more than a month	Yes	53	35%	5,776	5,038
	No	97	65%	4,887	6,308

**Table 6:** Average Annual Treatment Costs in US $ by DM Patient Complications in the UAE during 2004 (Weighted, N = 150)

	No complications	Microvascular Compliations	Macrovascular Complications	Both Macro + Microvascular Complications
**Average Annual Treatment Costs**	1,605	3,453	10,300	15,104
**Hospital Costs**	135	358	761	541
**Ratio**	0	2.654500726	5.646071947	4.016776899

Estimates and associations were not substantially affected by weighting cases (inversely) by frequency of clinic attendance, suggesting that our sampling method did not introduce substantial bias. As many of the factors considered here are probably correlated, we carried out analysis of covariance to identify which factors are independently associated with (natural logarithm of) costs. Possible sampling bias was corrected for by including the frequency of clinic attendance as an additional co-variable. Table (7) shows the results of this analysis. Variables not significantly correlated with the dependent variable were removed sequentially (i.e. backward selection) until only (marginally) significant variables remained in the model. Interestingly, type of diabetes (1 or 2) was not included as it was not significantly associated with costs after adjustment for treatment with insulin. Surprisingly, unlike a history of myocardial infarction which was associated with an exp (0.72) = 2.05 fold increase in costs, a history of stroke was not associated with significantly increased medical costs.

**Table 7:** Economic Costs of Diabetes Mellitus Parameter Estimates, analysis of covariance of factors affecting costs.

Parameter	Value	B	Std. Error	t	P-value
					
Intercept		8.089	.549	14.744	.000
History of Heart attack		.720	.147	4.905	.000
Foot/leg ulcer		.388	.140	2.765	.007
Age (years)		.012	.006	2.117	.036
Disease duration (years)		.024	.011	2.174	.031
Number of annual visits to DM clinic	None	-1.137	.308	-3.687	.000
	1-2	-1.042	.259	-4.028	.000
	3-6	-.565	.220	-2.572	.011
	7-11	-.137	.224	-.611	.542
	12+ (ref)	0(a)	.	.	.
Dyslipidemia	Yes	.842	.293	2.873	.005
	No	.423	.300	1.411	.161
	DK (ref)	0(a)	.	.	.
Use of insulin		.752	.155	4.840	.000
DM retinopathy	Yes	-.494	.349	-1.414	.160
	NO	-.660	.353	-1.871	.064
	DK (ref)	0(a)	.	.	.
Mode of diagnosis	Incidental	.578	.214	2.700	.008
	Screening	-.151	.161	-.935	.351
	Symptomatic(ref)	0(a)	.	.	.

Dependent Variable: (natural) logarithm of cost.

a This parameter is set to zero because it is redundant.

## Discussion

This is the first study assessing the costs of diabetes care in the UAE. Despite numerous limitations, the study was able to assess and analyze DM patients' management costs in the UAE and to present estimates that would hopefully help directing attention to the sizeable financial burden of the disease, for the first time in the UAE. To do that the study analyzed streams of DM patients' management costs, based on patients' profiles, for those without DM complications during one year. The study revealed that the average management costs per diabetic patient without complications in the UAE were $1,605 (SD ± $1,473) compared to $5,645 (SD ± $5,966) for those with DM complications. As expected costs were substantial but varied significantly with the initial mode of diagnosis, with the highest costs incurred by those diagnosed incidentally. Delay in diagnosis can directly increase complications and then lead to higher costs. Additionally, the presence of DM related complications, concomitant diseases such as hypertension, disease duration, age, insulin dependence and a history of myocardial infarction were shown to have multiplicative effect on patients' management costs. This clearly shows the impact of diabetes related late complications on the cost. Similar conclusions have been drawn by other studies [19,20].

Hospital admissions accounts for the largest part of diabetes cost, the extra need for inpatient hospital care for patients who have developed late complications will greatly affect cost since hospital bed-day has a relatively high unit cost compared with other resources and overall medication costs. Furthermore, most diabetics on oral hypoglycemic agents receive insulin soon after hospital admission for complications and this further increase the costs.

Our estimates appear to be of the same order of magnitude as those from Western countries, which seems logical as health care facilities in the UAE are of similar or even higher standard compared to those in many developed countries [13]. The comparison with developing countries in Asia and Africa is more difficult due to lack of information on patients' health care expenditures from most of these countries. However, where data are available they suggest -as expected - much lower levels of expenditure. For example, a recent study assessing the treatment costs of diabetics in Karachi - Pakistan [21] estimated the annual mean treatment costs per DM patient to be $197 only. Another example is a recent study from Iran in 2009, which gave an annual cost figure of US$ 152 per DM patient [9]. Similarly, in Tunisia, an analysis in 1994 estimated an annual cost figure as low as US$ 117 [10], in Egypt costs were even lower [11] and a recent study in Sudan showed direct costs to amount USD 175 per year [12]. Middle income countries, such as those in Latin America and the Caribbean region, tend to be in-between Western and developing countries [22]. Of course, treatment costs exclude many intangible costs which are also very high in developing countries. For example, the World Bank and WHO, together suggest that 80% of the annual intangible losses related to DM and its complications are incurred in developing countries [23].

A major limiting factor of our study is that it did not correct for costs that would have been incurred in the absence of DM. For example, some vascular complications and costs incurred by them may have occurred in the absence of DM. This may have led to some overestimation of costs. By contrast, DM may have aggravated conditions not considered in this study (e.g. hospitalization duration after accidents) which may have led to some underestimation of costs. However, by sampling from patients attending clinics, we may have oversampled frequent attendees. Although we tried to compensate for this using appropriate statistical technique such as re-weighting, we cannot be absolutely sure that this yields exactly the same results as a truly random sample. Thus, our estimates should be treated with caution.

## Conclusions

In view of the present high prevalence of DM in the UAE, and the potential costs associated with its management, investments in prevention are expected to be profitable and worthy. Likewise, the control of the concomitant risk factors for vascular complications, such as obesity, dislipidemia, and hypertension are key elements to reduce progression to these complications, and therefore, reduce the potential management costs associated with them. However, the high prevalence of incidental diagnosis among UAE patients, the primary source for the high DM treatment costs, suggest that diabetes may be under-diagnosed in the UAE population and tend to remain unrecognized until major complications occur. This suggests that the onset of the disease could be substantially reduced, and therefore the potential costs associated with it, if the high risk population is screened for DM on regular basis. However, to offset the potential costs of screening it may be worthy looking for other risk factors as well, such as obesity, hypertension or vascular diseases to help reducing their incidence, and therefore further reducing the overall burden of chronic diseases in the UAE.

## List of abbreviations

ADA: American Diabetes Association; DM: Diabetes Mellitus; ER: Emergency Room; EUR: EURO; GCC: Gulf Countries Citizens; IDF: International Diabetes Federation; PHCC: Primary Health Care Clinic; UAE: United Arab Emirates; $:US dollars

## Competing interests

The authors declare that they have no competing interests.

## Authors' contributions

FM and MS formulated the research question and participated in the design of the study. NN performed the statistical analyses and drafted the manuscript together with FM and MS. All authors contributed to the manuscript with their interpretation of results and comments on earlier drafts. All authors read and approved the final manuscript.

## Pre-publication history

The pre-publication history for this paper can be accessed here:

http://www.biomedcentral.com/1471-2458/10/679/prepub
